# Appropriateness and affordability of prescriptions to diabetic patients attending a tertiary hospital in Eastern Uganda: A retrospective cross-sectional study

**DOI:** 10.1371/journal.pone.0245036

**Published:** 2021-01-05

**Authors:** Samuel Baker Obakiro, Kenedy Kiyimba, Agnes Napyo, Andrew Marvin Kanyike, Wilberforce John Mayoka, Aishah Ggalabuzi Nnassozi, Beatrice Aguti, Gabriel Madut Akech, John Paul Waako

**Affiliations:** 1 Faculty of Health Sciences, Department of Pharmacology and Therapeutics, Busitema University, Tororo, Uganda; 2 Faculty of Health Sciences, Department of Public and Community Health, Busitema University, Tororo, Uganda; 3 Infectious Disease Clinic, Mbale regional referral Hospital Uganda, Mbale, Uganda; Qatar University, QATAR

## Abstract

**Background:**

Irrational prescription of drugs can lead to high cost of treatment thus limiting access to essential medicines. We assessed the affordability and appropriateness of prescriptions written for diabetic patients in Eastern Uganda.

**Methods:**

We collected secondary data from the health management information system registers of patients who attended the outpatient medical clinic at Mbale regional referral hospital from January 2019 to December 2019. The average cost of the prescriptions was calculated and adjusted odds ratios for predictors for unaffordability estimated using logistic regression. Computed scores for indicators of rational drug prescription were used to assess the extent of rational prescribing.

**Results:**

The median cost per prescription was USD 11.34 (IQR 8.1, 20.2). Majority of the diabetic patients (n = 2462; 94.3%, 95% CI: 93.3–95.1%) could not afford the prescribed drugs. Predictors for unaffordability were if a prescription contained: ≥ 4 medicines (AOR = 12.45; 95% CI: 3.9–39.7); an injectable (AOR = 5.47; 95%CI: 1.47–20.32) and a diagnosis of diabetes mellitus with other comorbidities (AOR = 3.36; 95%CI: 1.95–5.78). Having no antidiabetic drug prescribed was protective for non-affordability (AOR = 0.38; 95%CI: 0.24–0.61). The average number of drugs per prescription was 2.8. The percentage prescription of drugs by generic name and from the essential medicine and health supplies list of Uganda were (6160/7461; 82.6%, 96% CI: 81.7%-83.4%) and (6092/7461; 81.7%, 95% CI: 80.8%-82.5%) respectively against WHO standard of 100%.

**Conclusion:**

The majority of diabetic patients (94.3%) in Eastern Uganda cannot afford to buy prescribed medicines. The government should therefore ensure that essential medicines are readily accessible in public health facilities.

## Introduction

Diabetes mellitus (DM) is a chronic metabolic disorder associated with various complications and comorbidities such as peripheral vascular disease, coronary artery disease, hypertension, metabolic syndrome among others. By 2016, over 463 million people were diagnosed to have DM globally and in Uganda the prevalence was at 2.7% [1]. It was predicted that by 2035, the prevalence of DM in Uganda and many developing countries would have doubled [2]. The International Diabetes Federation (IDF) estimated that the number of people with DM in Africa will increase from 14.2 million in 2015 to about 34.2 million in 2040.This would translate to an increase in the global expenditure from the current $673 billion to about $802 billion assuming constant per capita healthcare expenditures [3]. The use of anti-diabetics and other drugs is an integral component in the management of DM and its associated comorbidities in many health care systems. However, the total availability of most anti-diabetic and anti-hypertensive drugs is usually low, especially in primary hospitals and in the absence of health insurance reimbursement in developing countries [4].

The World Health Organization (WHO) acknowledged that access to essential medicines in many developing countries is a serious challenge. Their health care systems are highly constrained by the limited essential medicine supplies and overwhelmingly high patient turn up [5,6].

In Uganda, 2 in 10 individuals diagnosed with a chronic disease such as DM actually had a medicine for that disease available at home [7]. This limited access to essential antidiabetic medicines in public health facilities cause patients to purchase these drugs from pharmacies, retail drug shops and private hospitals. However, studies have reported that many of the prescribed antidiabetic and antihypertensive medicines are unaffordable to the public in developing countries. For instance in Zambia, majority of surveyed antidiabetic and antihypertensive medicines were inadequately available (<80%) and most of them were unaffordable [8]. This is because apart from a significant number of people living below the poverty line in such countries, they have a high disease burden and inadequate access to basic needs of life. This results into non-adherence to treatment schedules and eventually poor treatment outcomes [9].

The WHO recommends that medicines are appropriately prescribed and dispensed while being used during diagnosis, prevention and treatment of diseases and this is what is referred to as “rational drug use”. Irrational prescription practices such as polypharmacy, over prescription of injectable and antibiotics, prescription of medicines outside the essential medicine list and not following standard treatment guidelines are common among health care systems in developing countries [10]. Inappropriate drug prescribing and dispensing is responsible for more than 50% wastage in expenditure on essential medicines. Irrationally prescribed drugs do not only increase patient and government expenditures but also may result into drug toxicities. Therefore, we undertook this study to evaluate the appropriateness and affordability of prescriptions for diabetic patients attending the medical special clinic at Mbale regional referral hospital, the largest public tertiary hospital in Eastern Uganda.

## Materials and methods

### Study sites and settings

This study was conducted at the Outpatient medical clinic of Mbale Regional Referral Hospital (MRRH) located in Mbale Municipality, Eastern Uganda. MRRH, one of the fourteen (14) regional referral (tertiary) hospitals in Uganda serves sixteen (16) surrounding districts of Eastern Uganda. These are Mbale, Budaka, Pallisa, Kibuku, Butebu, Butalejja, Tororo, Manafwa, Namisindwa, Bududa, Bulambuli, Sironko, Bukedea, Kapchorwa, Bukwa and Busia. The hospital has a total bed capacity of 548 and provides specialized health services to over five million people in Elgon region and even beyond. Besides hosting medical interns from the Ministry of Health, MRRH is also the major teaching hospital for surrounding medical and nursing schools including Busitema University Faculty of Health Sciences. The diabetic clinic is a specialized clinic hosted within the general outpatient medical clinic of the MRRH. An estimated 10,000 patients are managed by this clinic annually.

Uganda’s healthcare system is hierarchical in nature with chronologically increasing cadres of healthcare facilities. The lowest cadre is the Village Health Teams (VHTs), also known as a health centre HCI and predominantly offers health education, preventive and simple curative services in communities. The next level is HCII which offers out-patient services. Next in level is HCIII, which in addition to HCII services offers in-patient, simple diagnostic and maternal health services. Above HCIII is the HCIV which provides surgical services in addition to all the services provided at HCIII. Beyond HCIV we have the district hospitals. At the national level, there are national referral hospitals, regional referral hospitals and semi-autonomous institutions in respective hierarchy [11]. At all these cadres of health care, all prescribed medicines are provided to the patient at no cost regardless of being outpatients or inpatients.

However, due to the inadequate supply of essential medicine to public health facilities coupled with high patient turn up, public health care facilities usually suffer from prolonged drug stock outs [4]. Since many patients do not have health insurance coverage, they usually buy these essential medicines from private pharmacies and drug shops to access primary health care [11,16].

### Study design and population

This was a retrospective cross-sectional study that utilized secondary data from the health management information system (HMISFORM 031) registers of the outpatient medical clinic at MRRH. The study population was diabetic patients attending the outpatient medical clinic at MRRH. This population did not include paediatric patients because this nature of patients receive their care from the paediatric clinic. Neither did this study include pregnant women because these receive care from the antenatal clinic.

### Sample size and sampling procedure

We followed the WHO guidelines of including atleast 600 prescriptions while investigating medicine use in health facilities [12]. In this study, we used a total of 2612 prescriptions of diabetic patients that were sampled from the register of the outpatient medical clinic, MRRHfrom January 2019 to December 2019.On average, two hundred and twenty (220) observations were systematically randomly selected from each month and included in the study.

### Inclusion / Exclusion criteria

Observations (entries) with complete prescription data in the registers were extracted and analysed for affordability and rational prescribing. All prescriptions having DM as one of the diseases diagnosed were considered. Observations with illegible information were excluded from the study.

### Data collection

Research assistants with pharmacy training background were recruited to assist in data collection. The whole research team was then trained on the data collection process to minimise interpretation bias of data to be collected. A data collection tool was designed in Microsoft Excel (Microsoft Corporation, USA) to capture secondary data on different variables of each patient prescription entry as it appeared in the register. Retrospective data from January 2019 to December 2019 were then collected manually from handwritten registers between February 2020 and May 2020. Following entry, data was checked periodically for completeness by the research team. An observation was considered complete if it contained all the required variables of interest which included gender, age, location, disease diagnosis and drugs prescribed.

### Surveys and measures

The primary outcome of this study was affordability of the prescription. This was determined by calculating the total cost of the prescribed medicines in the prescription and computing the number of days it would take to pay off the cost based on the average income of people in Eastern Uganda. The total cost was obtained by summing up the individual costs of each drug in the prescription. The cost of each drug in the prescription was obtained by multiplying the total quantity of that drug with the average unit cost based on the average retail prices of the drugs in pharmacies and drug shops in Mbale district (S1 Table). The quantity of each drug prescribed was first calculated basing on the prescribed dose and frequency [12]. The average retail prices were calculated from a survey done regarding the unit cost of different drugs as sold from selected pharmacies and retail shops around Mbale town. The obtained price list is attached as a S1 Table. The calculated costs of prescriptions were compared with the average monthly income of lowest government paid servant as extracted from the Uganda National Household survey (UNHS) 2016/2017 [13]. From the UNHS 2016/2017 report, the average monthly income of lowest government employed person in 2019 was $44.5 (exchange rate 3704/ =). This on average translates into approximately $1.5 per day. Affordability of prescription was categorized into two levels. All prescriptions that required a maximum of three (3) days were collectively categorized as affordable and coded 0. The rest of the prescriptions that required more than three days were categorized as “unaffordable” and coded 1 [6]. Other variables were categorized as shown in Table 1 for comparison purposes. Prescribed medicines and diagnoses were classified according to the Anatomical Therapeutic Chemical (ATC) classification system and international system of classification of disease respectively.

**Table 1:** Demographics and characteristics of prescriptions for diabetic patients.

Characteristic	Total N = 2612 n (%)	Prescription not affordable N = 2462 n (%)	Prescription affordable N = 150 n (%)
**Age (years)**			
>40	2205 (84.4)	2077 (84.4)	128 (85.3)
30 to 40	333 (12.8)	314 (12.8)	19 (12.7)
<30	74 (2.8)	71 (2.8)	3 (2)
**Gender**			
Male	1611 (61.7)	1511 (61.4)	100 (66.7)
Female	1001 (38.3)	951 (38.6)	50 (33.3)
**Setting**			
Rural	1751 (67)	1653 (67.1)	98 (65.3)
Urban	861 (33)	809 (32.9)	52 (34.7)
**Diagnosis**			
Diabetes Mellitus alone	1660 (63.5)	1554 (63.1)	106 (70.7)
Diabetes Mellitus and Hypertension	602 (23.1)	582 (23.6)	20 (13.3)
Diabetes Mellitus with any other apart from Hypertension	202 (7.7)	187 (7.6)	15 (10)
Diabetes Mellitus, Hypertension and any Other	148 (5.7)	139 (5.7)	9 (6)
**Class of drugs prescribed**			
Biguanides	439 (16.8)	372 (15.1)	67 (44.7)
Sulfonylureas	21 (0.8)	17 (0.7)	4 (2.7)
Biguanides and sulfonylureas	1205 (46.1)	1184 (48.1)	21 (14)
Insulin	709 (27.1)	707 (28.7)	2 (1.3)
No antidiabetic	238 (9.1)	182 (7.4)	56 (37.3)
**Major classes of antibiotics prescribed**			
Penicillins	105 (4)	101 (4.1)	4 (2.7)
Cephalosporins	29 (1.1)	29 (1.2)	0 (0)
Fluoroquinolones	53 (2)	46 (1.9)	7 (4.7)
Tetracyclines	1 (0.04)	1 (0.04)	0 (0)
Aminoglycosides	8 (0.3)	8 (0.3)	0 (0)
Macrolides	17 (0.7)	16 (0.6)	1 (0.7)
Nitroimidazoles	40 (1.5)	33 (1.3)	7 (4.7)
2 antibiotic classes	59 (2.3)	53 (2.2)	6 (4)
3 antibiotic classes	4 (0.2)	4 (0.2)	0 (0)
No antibiotic	2296 (87.9)	2174 (83.2)	122 (81.3)

The secondary outcome variables were appropriateness of the prescriptions in reference to guidelines set by WHO in collaboration with the International Network of Rational Use of Drugs (INRUD). To assess this, indicators recommended by the WHO/INRUD were calculated. These are; (1) average number of medicines per prescription, (2) percentage encounter with antibiotics, (3) percentage of medicines prescribed by generic name, (4) percentage of injectable medicines in the medicines prescribed and (5) percentages of medicines prescribed from the Essential Medicine and Health Supplies List for Uganda (EMHSLU). These secondary outcomes were calculated using Eqs 1–5:

$$\mathrm{Average}\;\mathrm{number}\;\mathrm{of}\;\mathrm{medicines}\;\mathrm{per}\;\mathrm{prescription}=\frac{\mathrm{Total}\;\mathrm{number}\;\mathrm{of}\;\mathrm{medicines}\;\mathrm{prescribed}}{\mathrm{Total}\;\mathrm{number}\;\mathrm{of}\;\mathrm{prescriptions}}$$ Eq 1

$$Percentage\;of\;medicines\;prescribed\;by\;generic\;name=\frac{\mathrm{Total}\;\mathrm{number}\;\mathrm{of}\;\mathrm{drugs}\;\mathrm{prescribed}\;\mathrm{by}\;\mathrm{generic}\;\mathrm{name}}{\mathrm{Total}\;\mathrm{number}\;\mathrm{of}\;\mathrm{prescribed}\;\mathrm{drugs}}x\;100$$ Eq 2

$$Percentage\;of\;medicines\;prescribed\;from\;the\;EMHSLU=\frac{\mathrm{Total}\;\mathrm{number}\;\mathrm{of}\;\mathrm{medicines}\;\mathrm{prescribed}\;\mathrm{from}\;\mathrm{EMSHLU}}{\mathrm{Total}\;\mathrm{number}\;\mathrm{of}\;\mathrm{prescribed}\;\mathrm{drugs}}x\;100$$ Eq 3

$$Percentage\;of\;encounter\;of\;an\;antibiotic=\frac{\mathrm{Total}\;\mathrm{number}\;\mathrm{of}\;\mathrm{antibiotics}\;\mathrm{prescribed}}{\mathrm{Total}\;\mathrm{number}\;\mathrm{of}\;\mathrm{prescribed}\;\mathrm{medicines}}x\;100$$ Eq 4

$$Percentage\;of\;encounter\;of\;an\;injectable\;medicine=\frac{\mathrm{Total}\;\mathrm{number}\;\mathrm{of}\;\mathrm{injectable}\;\mathrm{medicines}}{\mathrm{Total}\;\mathrm{number}\;\mathrm{of}\;\mathrm{prescribed}\;\mathrm{medicines}}x\;100$$ Eq 5

The WHO prescription parameters were put in place to improve the appropriateness of prescriptions during patient care. An average number of two medicines per prescription are recommended to reduce polypharmacy. In an effort to curb antibiotic drug resistance, the percentage encounter with antibiotics per prescription should be less than 30%. Different brands of drugs exist on market, hence it is recommended to practice 100% generic prescribing, this ensures effective communication and information exchange amongst health care providers, additionally helps tame the cost of treatment that may be escalated by prices of the medicine brands. The use of injectable medicine is often associated with a number of challenges, some of which may include the need for trained personnel to administer the medicine, pain, nerve injury and potential exposure of a patient to infections hence these should make up less than 10% of the total prescriptions. Every country has an Essential Medicine and Health Supplies List; this entails a list of drugs that have been proved safe, efficacious and cost effective in that specific region, hence 100% of the drugs prescribed should be from that list.

### Data analysis

Data were entered into an Excel spread sheet by two independent data entrants and exported for analysis into STATA version 14.0 (StataCorp, College Station, Texas, USA). Continuous data were summarised into means and standard deviations if normally distributed. Otherwise, they were summarised into medians with interquartile ranges if not normally distributed. Categorical variables were presented as frequencies and proportions. The proportion of patients that could not afford the prescribed medicines was estimated and the confidence limits were calculated using the exact method. Multivariable logistic regression analysis was used to estimate the adjusted odds ratios of the independent variables on unaffordability of prescribed medicines while controlling for confounding. All variables with p<0.25 at the bivariate level were included in the initial model at the multivariate analysis. All variables with p<0.1 and those of biological or epidemiologic plausibility (from previous studies) were included in the second model.

### Ethical consideration

Ethical approval was obtained from CURE–Children’s Hospital Uganda Research and Ethics Committee (CCHU-REC/10/019), administrative clearance from Mbale regional referral Hospital and the Uganda National Council of Science and Technology (HS2686). A waiver of consent was applied for and granted by the Research and Ethics committee of MRRH to use the prescriptions records in this study. Patient confidentiality was ensured by giving a specific number code to each patient data instead of their names.

## Results

### Demographics and baseline characteristics of prescriptions

A total of 2612 prescriptions were included in the study. The mean age of patients for whom the prescriptions were made was 54.2 years (SD 12.6). Majority were male (n = 1611, 61.7%) and from a rural residence (n = 1751, 67%). More than half were suffering from only diabetes mellitus (n = 1660, 63.5%). The median cost per prescription was $11.34 (IQR 8.1, 20.2). The majority (n = 2462, 94.3%; 95% CI: 93.3–95.1%) could not afford the medicines that had been prescribed for them. Mostly oral anti-diabetics were prescribed (n = 1665, 63.7% of prescriptions). In 46.1% (n = 1205) of the prescriptions, biguanides were prescribed in combination with sulfonylureas. Antibiotics were prescribed in 12.1% (n = 316) of the prescriptions. The results are summarized in Table 1.

### Predictors for unaffordability

A number of prescription-related characteristics were associated with unaffordability (Table 2). The medicines prescribed were most likely to be unaffordable if the patient for whom the drugs were prescribed had been diagnosed with 2 or more chronic illnesses (Diabetes Mellitus and hypertension, AOR = 3.36; 95%CI: 1.95–5.78; Diabetes Mellitus, hypertension and any other disease, AOR = 2.28; 95%CI: 1.01–5.26) compared with those who suffered from 1 chronic illness. If the total number of medicines prescribed were 4 or more (AOR = 12.45; 95% CI: 3.9–39.7), the patient was most likely not to afford the prescribed medicines compared to if they were less than 4. If there was an injectable drug prescribed, the patients were most likely not to afford the prescription (AOR = 5.47; 95%CI: 1.47–20.32). Having no antidiabetic drug prescribed was protective for non-affordability (AOR = 0.38; 95%CI: 0.24–0.61) (Table 2).

**Table 2:** Predictors for non-affordability of medicines prescribed for diabetic patients.

Characteristic	Unadjusted odds ratio	Adjusted odds ratio
**Age (Years)**		
>40	1	1
30 to 40	1.02 (0.62–1.67)	0.76 (0.43–1.34)
<30	1.46 (0.45–4.69)	1.27 (0.34–4.75)
**Gender**		
Male	1	1
Female	1.26 (0.89–1.78)	1.1 (0.74–1.63)
**Setting**		
Rural	1	1
Urban	0.92 (0.65–1.3)	0.89 (0.6–1.32)
**Diagnosis**		
Diabetes Mellitus alone	1	1
Diabetes Mellitus and hypertension	1.98 (1.22–3.23)	**3.36 (1.95–5.78)**
Diabetes Mellitus, hypertension and any other disease	1.05 (0.52–2.12)	**2.28 (1.01–5.26)**
Diabetes Mellitus and any other disease apart from Hypertension	0.85 (0.48–1.49)	1.06 (0.55–2.06)
**Total number of drugs prescribed**		
<4 drugs	1	1
> or equal to 4 drugs	8.31 (3.87–17.83)	**12.45 (3.9–39.7)**
**Number of injectable drugs per prescription**		
None	1	1
1 or more injectable drugs	14.91 (5.5–40.41)	**5.47 (1.47–20.32)**
**Class of drugs prescribed**		
Biguanides	1	1
Sulfonylureas	0.77 (0.25–2.35)	0.72 (0.22–2.4)
Biguanides and sulfonylureas	10.15 (6.14–16.81)	**11.11 (6.64–18.61)**
Insulin	63.67(15.51–26.31)	**17.79 (2.95–107.28)**
No anti-diabetic	0.58 (0.39–0.87)	**0.38 (0.24–0.61)**

### Rational drug prescription performance indicators

The average number of medicines per prescription was 2.8 which was slightly above the WHO recommended value (2 medicines per prescription). The percentage encounter with an injectable medicine as well as antibiotic prescribed was within the WHO recommended standards. Although prescription of medicines by generic name and from the essential medicine and health supplies list of Uganda was less than 100%, the observed percentages (>80%) were good (Table 3).

**Table 3:** Performance of the facility as regards the WHO/INRUD indicators of rational drug use.

Indicator	WHO standard	Score of the diabetic clinic–MRRH
Average number of medicines per prescription	Should be less than 2	2.85 (SD 1.23)
Percentage of medicines prescribed by generic name	Should be 100%	(6160/7461;82.6%(95% CI: 81.7% -83.4%)
Percentage of medicines prescribed from the EMHSLU	Should be 100%	(6092/7461; 81.7%(95%CI: 80.8% - 82.5%)
Percentage of antibiotics in the prescribed medicines	Should be less than 30%	(381/7461; 5.1%, (95%CI: 4.6% - 5.6%)
Percentage of medicines prescribed which are injectable drugs	Should be less than 10%	(750/7461; 10.1%, (95%CI: 9.4% - 10.8%)

## Discussion

This study sought to assess the affordability and appropriateness of prescriptions given to diabetic patients at the outpatient medical clinic of MRRH. The majority of patients could not afford buying medicines prescribed to them. A plausible explanation for this could be the low income earned by these patients [13]. Besides the antidiabetic medicines being expensive especially insulin, the high cost of prescription observed was also attributed to the slightly higher average number of medicines prescribed per prescription (2.85). We observed a significant number of prescriptions (27.1%) with insulin being prescribed. Unaffordability of prescriptions due to irrational prescription practices has been reported in in India and Sierra Leone where average costs of prescriptions were $4.74 and $6.78 respectively [6,14]. Most prescriptions (46.1%) had a combination of two oral antidiabetic medicines prescribed: metformin (biguanide) and glibenclamide (sulfonylurea). In developing countries, there is limited opportunity for early intensive therapeutic intervention because many patients are usually diagnosed of the disease late, mostly after manifestation of life threatening complications [15]. Hence, the use of metformin alone as the first-line medication for management of type 2 diabetes may be inadequate in achieving optimal blood glucose levels. This therefore calls for combination therapy to achieve optimal therapeutic outcomes [16]. As much as metformin has a good safety profile with regards to hypoglycaemia, glibenclamide and other second-generation sulfonylureas are more potent hence given together to enhance the therapeutic benefit. Additionally, a combination of metformin and glibenclamide (oral) is both easy to administer and affordable to most patients [17]. Several studies have reported a similar trend in the prescription of anti-diabetics with biguanides and sulfonylureas being the most frequently prescribed [18–20]. High insulin prescription could have been aimed at controlling the abnormally high glucose levels in lately diagnosed patients who fail to respond well to the oral hypoglycaemic. However, insulin preparations are expensive, and therefore irrational prescription makes them unaffordable to the patients. DM comorbidities require different medications other than antidiabetic medicines to be managed. Thus, the other medicines also contribute to the high cost of prescription. In this study, anti-hypertensives (34.6%), vitamin B complex (16.6%), analgesics (10.8%), antibiotics (5.1%), steroids (0.6%) and anti-cholesterol drugs (1.2%) were frequently prescribed along with antidiabetic medicines.

Regarding the performance of the diabetic clinic in reference to the WHO indicators of rational use of medicines, the average of2.85 medicines per prescription was slightly higher than the optimal value of less than 2 medicines per prescription as recommended by WHO. A similar finding was previously reported in Western Uganda [21] where the average number of medicines per prescription was 2.6. This therefore would indicate presence of polypharmacy at the medical clinic of MRRH. However, due to the comorbidities associated with DM which requires multiple medications, the observed value of 2.85 is acceptable [22]. In Nepal, an average of 3.76 medicines per prescription was reported where67% prescriptions contained more than 5 medicines [23].

The percentage of medicines prescribed by generic name (82.6%) was slightly less than the recommended value (100%). Generic prescribing not only reduces the cost of treatment for the patient but also enables better information exchange and allows better communication among health care providers [24]. Our findings resonate with some previous studies. For instance in a study to assess the prescription pattern among DM type II inpatients in India, the percentage of medicines prescribed by their generic name were 76% [23]. Similarly, in Sierra Leone, it was 71.0% [14]. Henceforth, more emphasis should be put on continuous medical education and trainings by the regulatory bodies and frequent monitoring mechanism by medicine therapeutic committees to ensure complete generic prescribing standards.

In our study, 81.7% of medicines were prescribed from the Essential Medicines and Health Supplies List of Uganda (EMHSLU) which is slightly lower than the WHO recommended value of 100%. Previously, 79% of the medicines were prescribed from the EMHSLU in a tertiary hospital in Western Uganda. Non-adherence to prescribing from the EMHSLU could probably be due to the influence of medicine promoters in the region who persuade the prescribers using incentives to prescribe medicines which are not in the National list. Prescription from EMHSLU ensures patients access to safe, efficacious and cost effective medicines and hence should be adhered to at all times.

The percentage of antibiotic encounter among the prescriptions was 5.11% and this lies within the WHO recommended standard of less than 30%. This attributed to the antimicrobial stewardship program in the hospital has increased awareness about antibiotic use and misuse among health care professionals. The percentage of encounters with an injectable medicine prescribed was at the cut-off recommended by the WHO. This could be attributed to the fact that majority of the patients were suffering from DM type 2 which is largely managed using oral hypoglycaemic but also the nature of the clinic setting being outpatient. However, the observed proportion was contributed by insulin mixtard which is given as an injection and other injectable drugs for comorbidities. Injectable drugs are not only expensive but also require trained personnel to administer them. Irrational use of injections may expose a patient to unnecessary pain, promote the spread of infections like HIV/AIDS, hepatitis, and promote microbial resistance, muscle contractures and nerve injury [25]. From our study, prescriptions give to the diabetic patients in the outpatients department, MRRH are unaffordable, we therefore recommend the Ugandan government to ensure that these essential medicines are readily available and accessible in public health facilities so as to reduce on the mortality and morbidity associated with DM.

### Strengths and limitations

To the best of our knowledge this is the first study to report about affordability of prescriptions for DM patients in Eastern Uganda. With cases of DM increasing not only globally but also in Uganda, this study provides evidence to government and other stake holders the required evidence to make antidiabetic medicines and other medications for DM associated comorbidities readily available and accessible in public health facilities. Compliance to the standards of rational prescribing by prescribers at the medical clinic have also been reported for the first time.

Our study had some limitations too. We used secondary data from the HMIS registers and there might be alteration in the data on the actual prescription during entry into the HMIS registers. This was mitigated by ensuring that only observations with complete records are used in this study and checking for consistence in data entry by the records department at the tertiary hospital before data collection. We did not include some socio-demographic variables in the analysis of the data. The registries from which this data were extracted are not designed to capture some key socio-demographic information like patient employment, level of education and income earned. Therefore, we did not include this information as potential exposures for our outcome. We did not categorise insulin therapies into its different types because one of the categories had very few observations that would not have been representative to draw conclusions.

We further recommend a prospective cohort study to be done on the subject matter because the information in the registries had limitations and lacked important exposure variables that could have further informed our findings.

### Conclusions

Diabetic patients in Eastern Uganda cannot afford to buy prescribed medicines from private pharmacies and retail drug shops. A prescription was most likely not to be affordable if it contained more than three (3) medicines, an injectable medicine and DM with comorbidities. On the other hand, there was good compliance to rational prescribing practices at the medical clinic of the tertiary hospital.

## Supporting information

S1 Table Average prices of common drugs as sold from pharmacies and drug shops around Mbale town. (DOCX)

S1 Dataset (XLSX)
