# Supplementary material for: Appropriateness and affordability of prescriptions to diabetic patients attending a tertiary hospital in Eastern Uganda: A retrospective cross-sectional study
Source: PLoS One. 2021 Jan 5;16(1):e0245036. doi: 10.1371/journal.pone.0245036 (PMC7785215; doi:10.1371/journal.pone.0245036)
Supplement: S1 Table — (DOCX) [file pone.0245036.s001.docx]

**S1 Table: Average prices of common drugs as sold from pharmacies and drug shops around Mbale town**

|  | **Drug/strength** | **Form** | **Unit** | **Unit price (UgX)** |
| --- | --- | --- | --- | --- |
| 1 | Actrapid IM | vial | 1 | 25000 |
| 2 | Adalat 30mg [Nifedipine] | Tab | 1 | 700 |
| 3 | Albendazole 400mg | Tab | 1 | 1000 |
| 4 | Amlodipine 10mg | Tab | 1 | 700 |
| 5 | Amlodipine 5mg | Tab | 1 | 400 |
| 6 | Amlozaar | Tab | 1 | 1500 |
| 7 | Amoxycillin 250mg | Cap | 1 | 100 |
| 8 | Ampiclox 500mg | Cap | 1 | 200 |
| 9 | Aprazole powder | sachet | 1 | 2000 |
| 10 | Artemether/Lumefantrine (20mg/120mg) | Tab | 1 | 150 |
| 11 | Ascoril Syrup | Syr | 1 | 8000 |
| 12 | Atenolol 100mg | Tab | 1 | 150 |
| 13 | Atenolol 50mg | Tab | 1 | 100 |
| 14 | Atorvastatin 10mg | Tab | 1 | 300 |
| 15 | Atorvastatin 20mg | Tab | 1 | 600 |
| 16 | Azithromycin 500mg | Tab | 1 | 1500 |
| 17 | Bendroflumethiazide 5mg | Tab | 1 | 100 |
| 18 | Betapyn | Tab | 1 | 1000 |
| 19 | Bisoprolol 10mg | Tab | 1 | 1000 |
| 20 | Bisoprolol 2.5mg | Tab | 1 | 500 |
| 21 | Bisoprolol 5mg | Tab | 1 | 700 |
| 22 | Calcium Lactate | Tab | 1 | 100 |
| 23 | Captopril 25mg | Tab | 1 | 200 |
| 24 | Carbamazepine 100mg | Tab | 1 | 100 |
| 25 | Carbamazepine 200mg | Tab | 1 | 100 |
| 26 | Carbidopa/Levodopa | Tab | 1 | 500 |
| 27 | Cardilor 200 [Amiodarone] | Tab | 1 | 1000 |
| 28 | Cardiac Aspirin 75mg | Tab | 1 | 150 |
| 29 | Carvedilol 12.5mg | Tab | 1 | 900 |
| 30 | Carvedilol 3.125mg | Tab | 1 | 400 |
| 31 | Carvedilol 6.25mg | Tab | 1 | 600 |
| 32 | Ceftatime SB 1.5g IM [Ceftriaxone/Sulbactam] | vial | 1 | 5000 |
| 33 | Celecoxib 100mg (Zycel) | Tab | 1 | 300 |
| 34 | Celecoxib 200mg | Tab | 1 | 500 |
| 35 | Ciprofloxacin 500mg | Tab | 1 | 200 |
| 36 | Citirizine 10mg | Tab | 1 | 100 |
| 37 | Clopidogrel | Tab | 1 | 1500 |
| 38 | Clotrimazole cream | tube | 1 | 2500 |
| 39 | Cyrinol Syrup | Syr | 1 | 15000 |
| 40 | Daroxine [Cefuroxime 500mg] | Tab | 1 | 2000 |
| 41 | Diclofenac 50mg | Tab | 1 | 50 |
| 42 | Digoxin | Tab | 1 | 300 |
| 43 | Digoxin IM | amp | 1 | 1000 |
| 44 | Doxycycline 100mg | Cap | 1 | 100 |
| 45 | Enalapril 10mg | Tab | 1 | 600 |
| 46 | Enalapril 5mg | Tab | 1 | 400 |
| 47 | Esomeprazole 20mg | Tab | 1 | 300 |
| 48 | Esomeprazole 40mg | Tab | 1 | 500 |
| 49 | Euglim 4mg [Glimepride] | Tab | 1 | 500 |
| 50 | Ferrous Sulphate | Tab | 1 | 100 |
| 51 | Flexilor SR [Lornoxicam] | Tab | 1 | 1000 |
| 52 | Fluconazole 100mg | Tab | 1 | 1000 |
| 53 | Fluconazole 200mg | Cap | 1 | 1000 |
| 54 | Folic Acid | Tab | 1 | 50 |
| 55 | Furosemide 40mg | Tab | 1 | 100 |
| 56 | Furosemide IM | amp | 1 | 1000 |
| 57 | Ginsomin | Tab | 1 | 1000 |
| 58 | Glibenclamide 5mg | Tab | 1 | 100 |
| 59 | Gofen (Ibuprofen 400mg) | Cap | 1 | 1000 |
| 60 | Go-Gel cream | Tube | 1 | 4000 |
| 61 | Gramocef 400mg (Cefixime) | Tab | 1 | 4000 |
| 62 | Hepaticum | Cap | 1 | 1000 |
| 63 | Hydroxychloroquine | Tab | 1 | 1500 |
| 64 | Lactulose Syrup | Syr | 1 | 10000 |
| 65 | Levobact 500 [Levofloxacin] | Tab | 1 | 2000 |
| 66 | Levobact 750 | Tab | 1 | 3500 |
| 67 | Levothyroxine 100mg | Tab | 1 | 1200 |
| 68 | Livolin | Cap | 1 | 1000 |
| 69 | Lorazepam | Tab | 1 | 300 |
| 70 | Losartan 50mg | Tab | 1 | 900 |
| 71 | Losartan-H [+ Hydrochlorthiazide] | Tab | 1 | 800 |
| 72 | Mediven cream [Betamethasone] | tube | 1 | 3000 |
| 73 | Metformin 500mg | Tab | 1 | 100 |
| 74 | Metronidazole 200mg | Tab | 1 | 50 |
| 75 | Mixtard IM | vial | 1 | 25000 |
| 76 | Mupirocin cream | Tube | 1 | 18000 |
| 77 | Myprodol | Cap | 1 | 500 |
| 78 | NAT B | Cap | 1 | 1000 |
| 79 | Nebivolol 10mg | Tab | 1 | 1600 |
| 80 | Nebivolol 5mg | Tab | 1 | 1300 |
| 81 | Neurotone | Tab | 1 | 1000 |
| 82 | Neutraflux Syrup | Syr | 1 | 12000 |
| 83 | Nifedipine 20mg | Tab | 1 | 100 |
| 84 | Nitroglycerin | Tab | 1 | 300 |
| 85 | Omeprazole 20mg | Cap | 1 | 100 |
| 86 | Oramin | Tab | 1 | 500 |
| 87 | Ornilox | Tab | 1 | 3500 |
| 88 | Osteomin | Tab | 1 | 1500 |
| 89 | Paracetamol 500mg | Tab | 1 | 50 |
| 90 | Phenobarbitone | Tab | 1 | 100 |
| 91 | Phenytoin | Cap | 1 | 150 |
| 92 | Prednisolone | Tab | 1 | 50 |
| 93 | Pregabalin 75mg | Cap | 1 | 1500 |
| 94 | Pioglitazone | Tab | 1 | 500 |
| 95 | PyloKit | Tab | 1 | 35000 |
| 96 | Rabeloc 20mg [Rabeprazole] | Cap | 1 | 1000 |
| 97 | Ranolazine | Tab | 1 | 300 |
| 98 | Relcer gel 180ml | Syr | 1 | 10000 |
| 99 | Renerve Plus | Tab | 1 | 2000 |
| 100 | Rosuvastatin 10mg | Tab | 1 | 600 |
| 101 | Rosuvastatin 20mg | Tab | 1 | 900 |
| 102 | Salbutamol 4mg | Tab | 1 | 50 |
| 103 | Salbutamol Inhaler |  | 1 | 10000 |
| 104 | Sensur tube | Tube | 1 | 10000 |
| 105 | Stednac | Tab | 1 | 1000 |
| 106 | Stednac gel | Tube | 1 | 10000 |
| 107 | Telmisartan-H 52.5mg | Tab | 1 | 900 |
| 108 | Telmisartan-H 92.5mg | Tab | 1 | 1200 |
| 109 | Toraxin [Cryptoheptadine] | Tab | 1 | 100 |
| 110 | Ubinext | Tab | 1 | 1500 |
| 111 | Valazyd-H [Valsartan/Hydrochlorthiazide] | Tab | 1 | 1000 |
| 112 | Vitamin A | Cap | 1 | 1000 |
| 113 | Vitamin B Complex | Tab | 1 | 50 |
| 114 | Vitamin C | Tab | 1 | 50 |

IM: Intramuscular, Cap: Capsule, Tab: Tablet, Syr: Syrup
